# The Effect of Reviewers’ Self-Disclosure of Personal Review Record on Consumer Purchase Decisions: An ERPs Investigation

**DOI:** 10.3389/fpsyg.2020.609538

**Published:** 2021-01-08

**Authors:** Jianhua Liu, Zan Mo, Huijian Fu, Wei Wei, Lijuan Song, Kewen Luo

**Affiliations:** ^1^School of Management, Guangdong University of Technology, Guangzhou, China; ^2^MBA School, Guangdong University of Finance and Economics, Guangzhou, China

**Keywords:** review record, self-disclosure, purchase decision, event-related potentials, N400, LPP

## Abstract

Personal review record, as a form of personally identifiable information, refers to the past review information of a reviewer. The disclosure of reviewers’ personal information on electronic commerce websites has been found to substantially impact consumers’ perception regarding the credibility of online reviews. However, personal review record has received little attention in prior research. The current study investigated whether the disclosure of personal review record influenced consumers’ information processing and decision making by adopting event-related potentials (ERPs) measures, as ERPs allow for a nuanced examination of the neural mechanisms that underlie cognitive processes. At the behavioral level, we found that the purchase rate was higher and that the reaction time was shorter when the review record was disclosed (vs. when it was not), indicating that the disclosed condition was more favorable to the participants. Moreover, ERPs data showed that the disclosed condition induced an attenuated N400 component and an increased LPP component relative to the undisclosed condition, suggesting that the former condition gave rise to less cognitive and emotional conflict and to more positive evaluations. Thus, by elucidating potential cognitive and neural underpinnings, this study demonstrates the positive impact of reviewers’ disclosure of personal review record on consumers’ purchase decisions.

## Introduction

In the current era of electronic commerce, online consumer reviews (OCRs) serve as principle cues for consumer decision making and attract much scholarly attention (Reyes-Menendez et al., 2019b). Previous work on OCRs has documented that the credibility of OCRs positively affects consumer purchasing of recommended products (Cheung et al., 2009; Yan et al., 2016, 2018; Grewal and Stephen, 2019). To lower the risks of purchasing products on electronic platforms, consumers generally resort to OCRs with a high level of credibility when making purchase decisions (Riley et al., 1954; Mcknight and Kacmar, 2006; Park et al., 2014). However, given that OCRs are posted online by strangers in most cases and that large volumes of OCRs are available, it is challenging for consumers to assess the credibility of OCRs (Metzger, 2007; Cheung and Thadani, 2012; Park et al., 2013; Reyes-Menendez et al., 2019a). Consequently, consumers have to exploit online informational cues in order to make credibility evaluations.

Several reviewer factors have been confirmed to influence the credibility and helpfulness of OCRs, including reviewer ranking (Baek et al., 2012), reviewer cumulative helpfulness (Cao et al., 2011), reviewer reputation (Racherla and Friske, 2012) and reviewer personal information disclosure (Xie et al., 2011). Among these factors, reviewer self-disclosure is an intriguing issue that has received increasing levels of attention from academics and practitioners. Self-disclosure is generally defined as “any information about himself which Person A communicates verbally to a Person B” (Riley et al., 1954; Cozby, 1973). In computer-mediated contexts, posting one’s personal information online is a typical means of self-disclosure. With the aid of personally identifiable information, a consumer is able to distinguish a reviewer from others online (Tidwell and Walther, 2002). In fact, it has been established that reviewers’ self-disclosure has a positive impact on consumers’ perception of source credibility, which in turn shapes consumer willingness to accept certain messages as well as their willingness to buy the product (Forman et al., 2008; Cox et al., 2009; Yoo et al., 2009; Xie et al., 2011). For example, Xie et al. (2011) reported that the presence of online reviewers’ personally identifiable information positively affect consumers’ perceived credibility of ambivalent online hotel reviews and hotel booking intentions.

Extant OCRs-related research has identified reviewers’ names, geographic locations, interests and profile pictures as main types of personal information (Forman et al., 2008; Xie et al., 2011). Some online shopping websites, such as Amazon.com, also display the reviewers’ personal review records alongside with other personal information. Personal review record, also known as personal review history, refers to the entire past review information of a reviewer. If a reviewer chooses to disclose his (or her) personal review record, other consumers are able to see all the product reviews he has posted before. Though prior research has endeavored to seek out how the disclosure of personally identifiable information (e.g., profile picture, name and geographic location) affects perceived source credibility and consumer purchase decision (Lee and Shin, 2014; Xu, 2014; Liu and Park, 2015; Karimi and Wang, 2017), the disclosure of personal review record, however, has received little attention. Hence the present work is aimed to uncovering the effect of the disclosure of personal review record on consumers’ responses.

Information signaling theory, which has been applied to elucidate how consumers rely on various signals to form expectations about quality, is helpful in understanding the abovementioned effect. In online environment, the quality of products and services are generally difficult to evaluate due to information asymmetry (Akerlof, 1970). Signaling theory provides a framework to understand the various types of signals that are used to reduce information asymmetry and the situations in which they are used (Mavlanova et al., 2012). According to signaling theory, signals are observable and alterable attributes which can be used by individuals or organizations to communicate hidden or limited quality information to consumers to promote a purchase or transaction (Wells et al., 2011). Signals are particularly important in online contexts, because online contexts generally involve a higher level of uncertainty and risk than offline contexts (Mitra and Fay, 2010). Since OCRs are posted mostly by strangers online, it is difficult for potential consumers to assess the credibility of OCRs. Thus, consumers resort to any signals about the reviewer to aid their assessment of source credibility and message quality (Naujoks and Benkenstein, 2020). For instance, Le et al. (2018) demonstrated that source expertise signal was positively associated with perceived message quality. In the current study, the disclosure of personal review record might be seen by the potential consumers as a signal sent by the reviewer to show that his reviews are open to social scrutiny and are of high quality and credibility (Kirmani and Rao, 2000). Additionally, the disclosure of personal review record might also signal that the reviewer’s identity could be distinguished from the others in the online context. Hence we assumed that it would result in a notable increase in consumer behavioral intention when the reviewer’s personal review record was disclosed (vs. not disclosed). To the best our knowledge, this is the first study to conceptualize personal review record as a signal that influences consumer decision.

In recent years, the rapid advance in neuroscience has made it possible to incorporate event-related potentials (ERPs) technique into marketing related research. Changes in electrophysiological brain signals have been demonstrated to be useful for examining the perceptual and cognitive processes that occur in response to marketing stimuli (Ma et al., 2018). As a result, ERPs were adopted to examine the effect of the disclosure of personal review record on consumer purchase decision making. In the experiment, personal review record was set as either visible or invisible. Specifically, in the disclosed condition, an information cue indicating the disclosure of personal review record was provided whereas in the undisclosed condition, an information cue indicating the nondisclosure of personal review record was provided. In line with previous studies on consumer decision making, we mainly focused on N400 and late positive potential (LPP) components (Wang et al., 2015; Goto et al., 2017; Jin et al., 2017).

Generally, N400 is a negative deflection that mainly arises at approximately 400 ms post stimulus presentation in the frontal and central areas of the brain (Steffensen et al., 2008; Chen et al., 2010; Wang et al., 2015). Although N400 component is traditionally conceptualized as an indicator of semantic violations, it is also suggested to be an indicator of non-semantic conflict by recent research (Chen et al., 2010; Huang et al., 2014). In fact, semantic conflict can be viewed as a special case of informational conflict (Wang et al., 2015). It has been established that when a stimulus provides varied conflict information, a salient N400 component might be elicited, which suggests the occurrence of a phase of conflicting information processing when consumers are faced with incongruent extrinsic cues (Ma et al., 2014; Wang et al., 2015). In the current study, subjects might face more uncertainty regarding the authenticity of the product review in the undisclosed condition, which may denote the occurrence of higher levels of cognitive and emotional conflict when consumers are making purchase decisions. Therefore, we expected the undisclosed condition to induce a more negative N400 than the disclosed condition.

Belonging to the P300 family, LPP is a positive-going component that typically peaks at roughly 600 ms after the stimulus onset and lasts for a long duration (Schupp et al., 2000). LPP is widely distributed from the anterior regions to the posterior regions. Past research consistently suggests that the LPP component indicates the allocation of attentional resources to stimuli (Ito and Cacioppo, 2000; Langeslag et al., 2007; Qin and Han, 2009; Wang et al., 2015). LPP is also indicative of the evaluative categorization process before a final purchase decision is made, such that a more pronounced LPP amplitude would be induced by a more desirable stimulus than a less desirable one (Wang et al., 2015; Jin et al., 2017). In the current study, participants exposed to different extrinsic cues might also undergo an evaluative process of categorization. In contrast to the undisclosed condition, the disclosed condition was more preferable for the subjects because it signaled a higher level of credibility. Hence, we hypothesized that the disclosed condition would elicit an enlarged LPP amplitude relative to the undisclosed condition.

## Materials and Methods

### Participants

Thirty-five right-handed undergraduate students were recruited as paid volunteers. All participants had either normal or corrected-to-normal visual acuity and did not have any history of neurological or psychiatric disorders. The experiment was approved by the Internal Review Board of the Laboratory of Neuromanagement and Decision Neuroscience of Guangdong University of Technology. In accordance with the Declaration of Helsinki, written informed consent was obtained from each participant before the experiment. Data from two participants were discarded due to excessive artifacts, leaving thirty-three valid participants (16 females) aged 17–24 (*M* ± *SD* = 19.15 ± 1.25). A power analysis was performed to determine the sample size prior to the experiment. The suggested sample size was 14 when we assumed the effect size (f) to be 0.2 and the error probability (α) to be 0.05. Thus, the sample size of the current study fully met the requirement.

### Stimuli

Eighty T-shirts with similarly attractive appearance were selected from JD.COM, one of the most popular online B2C websites in China. Those 80 pictures were randomly assigned to the disclosed condition and the undisclosed condition. Twenty-two respondents who didn’t participate in the formal experiment were asked to rate the attractiveness, familiarity and complexity of each T-shirt on seven-point Likert scales ranging from 1 (very low) to 7 (very high). Paired *t*-tests showed that the attractiveness [M_disclosed_ = 4.50, *SD* = 0.67; M_undisclosed_ = 4.62, *SD* = 0.85; *t*(21) = −1.014, *p* = 0.322], familiarity [M_disclosed_ = 5.35, *SD* = 0.79; M_undisclosed_ = 5.31, *SD* = 0.81; *t*(21) = 0.660, *p* = 0.516] and complexity [M_disclosed_ = 3.38, *SD* = 0.46; M_undisclosed_ = 3.42, *SD* = 0.41; *t*(21) = −0.75, *p* = 0.462] did not differ between the disclosed condition and the undisclosed condition. Moreover, a group interview of students at Guangdong University of Technology was held to identify product ratings that were thought to be acceptable when purchasing a product. Product ratings refer to the aggregated review ratings computed according to all review ratings posted by consumers who have purchased the product. The five star rating system is widely used by electronic commerce websites, with one star (corresponding to 1.0) signaling the lowest score and five star (corresponding to 5.0) signaling the highest score. The result revealed that product ratings ranging between 4.1 and 5.0 were acceptable. Therefore, each product was paired with a product rating between 4.1 and 5.0, while the ratings remained consistent across experimental conditions. The disclosure of personal review record was manipulated by using information cues. In the disclosed condition, the cue “visible” was provided to indicate the disclosure of personal review record; while in the undisclosed condition, the cue “invisible” was provided to indicate the nondisclosure of personal review record. There were 40 trials in each condition and 80 trials in total.

### Procedure

**FIGURE 1:**
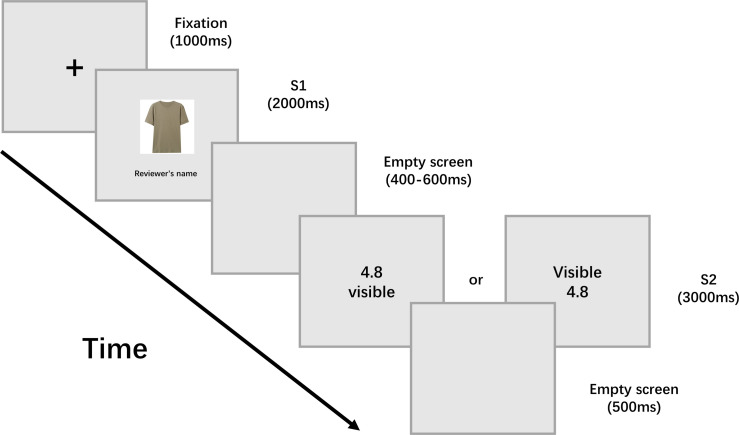
Experimental procedure. Participants were asked to make a purchase decision after viewing the cue information (S2).

During the experiment, participants were comfortably seated in a dimly lit and sound proof room. The experimental procedure was introduced on paper handouts and exemplars of detailed personal review records were shown to the participants. Each participant completed eight practice trials to become familiar with the task before the formal experiment. As is shown in Figure 1, each trial began with a fixation cross appearing in the center of a screen for 1,000 ms, which was followed by the presentation of an image of a T-shirt (S1, 4.5° × 6.0° visual angle) for 1,000 ms. This was followed by an interval with a random duration of 400–600 ms, after which a stimulus containing both product rating and personal review record information (S2, 2.1° × 2.4° of visual angle) was shown for 3,000 ms. The participants were asked to determine whether to buy the product as soon as possible within 3,000 ms. Keypads were used to provide responses and response-to-hand assignments were counterbalanced across the participants. To eliminate the potential influence of reading order, the relative positioning of product ratings and personal review record cues was counter-balanced. S2 was then followed by an inter-trial interval of 800 ms. There were four blocks and the ordering of trials was randomized within each block. Stimulus presentation and behavioral data recording were controlled using E-Prime 2.0 software (PST, Psychology Software Tools Inc.). The EEG experiment took about 15 min, including break time between blocks.

After completing the EEG experiment, the participants were asked to rate the displayed product offerings in terms of perceived disclosure, perceived trustworthiness (Flanagin and Metzger, 2016), and purchase intention (Dou et al., 2012). All ratings were made on seven-point scales (1 = “strongly disagree” to 7 = “strongly agree”).

### Electroencephalogram (EEG) Data Acquisition and Analysis

The electroencephalograms of the participants were recorded with an eego amplifier (ANT Neuro, Enschede, Netherlands) with a 500 Hz sampling rate and 0.1–100 Hz bandpass. An elastic electrode cap with 64 Ag/AgCl electrodes was used and the impedances of the electrodes were maintained at below 10 kΩ throughout the experiment. A cephalic electrode placed between FPZ and FZ served as a ground electrode. The left mastoid was used as an online reference. The EEG was re-referenced offline to the average of the left and the right mastoids.

ASALab 4.10.1 software (ANT Neuro, Enschede, Netherlands) was used to process offline EEG data. An eye movement correction algorithm was used to identify and correct Ocular artifacts. Before the ERPs data were segmented into epochs, a low-pass filter at 30 Hz (24 dB/Octave) was used to filter the raw data. The epochs of the stimulus were set to 1,000 ms, with 200 ms before the stimulus onset serving as the baseline and with 800 ms occurring post-stimulus onset. Trials involving amplifier clipping, bursts of electromyography activity, or peak-to-peak deflection exceeding ± 100 V were excluded from averaging. The EEG epochs were averaged for each participant within each condition and then grand averaged. Finally, data were analyzed using within-subjects repeated-measures ANOVAs.

Based on the processed data and past research on purchase decision making (Wang et al., 2015), two ERPs components, N400 and LPP, were analyzed in this study. Six electrodes (F3, FZ, F4, FC3, FCZ, and FC4) distributed from the frontal to fronto-central regions were selected for N400 analysis. Nine electrodes (F3, FZ, F4, FC3, FCZ, FC4, C3, CZ, and C4) distributed from the frontal to central regions were selected for LPP analysis. The mean amplitude of the N400 in the time window of 445–465 ms after the onset of S2 was used in a 2 (disclosure: disclosed and undisclosed) × 6 (electrodes: F3, FZ, F4, FC3, FCZ, and FC4) repeated-measure ANOVA. Similarly, the mean amplitude of LPP in the time window of 500–650 ms was used in a 2 (disclosure: disclosed and undisclosed) × 9 (electrodes: F3, FZ, F4, FC3, FCZ, FC4, C3, CZ, and C4) repeated-measure ANOVA. The Greenhouse–Geisser correction (Greenhouse and Geisser, 1959) was applied when the sphericity assumption did not apply (uncorrected dfs and corrected *p*-values were reported).

## Results

### Behavioral Results

**FIGURE 2:**
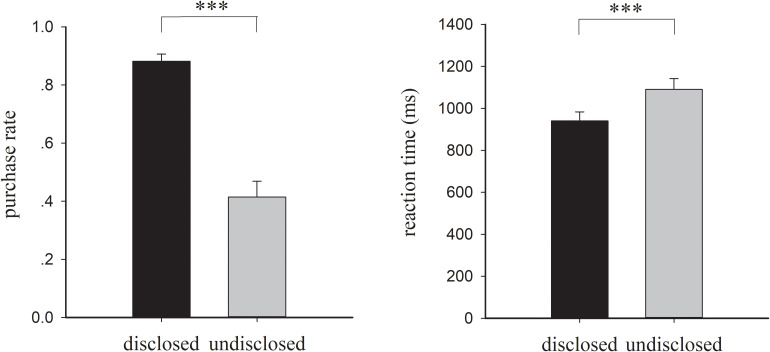
Behavioral results. The purchase rate and reaction time for each condition are shown. Error bars denote the standard error of the mean. ^∗∗∗^*p* < 0.001.

The one-way repeated measure ANOVA on purchase rate revealed a significant main effect of disclosure [*F*(1, 32) = 62.990, *p* < 0.001, η*_*p*_*^2^ = 0.663]. As illustrated in Figure 2, the purchase rate for the disclosed condition (*M* = 0.88, *SE* = 0.14) is higher than that for the undisclosed condition (*M* = 0.41, *SE* = 0.11). Furthermore, the effect of disclosure on reaction time was also significant [*F*(1, 32) = 32.803, *p* < 0.001, η*_*p*_*^2^ = 0.506]. The disclosed condition led to a significantly shorter reaction time (*M* = 940.45 ms, *SE* = 247.55) than the undisclosed condition (*M* = 1,090.40 ms, *SE* = 294.85). Statistical analysis results for ratings collected after the EEG experiment are shown in Table 1. Table 1 shows that perceived disclosure, trustworthiness and purchase intention were significantly higher for the disclosed condition than for the undisclosed condition.

**TABLE 1:** Statistical analysis results of the rated items.

Variables	Disclosed condition		Undisclosed condition		*F*	*p*
	*M*	*SE*	*M*	*SE*		
Perceived disclosure	5.64	0.69	2.24	0.93	680.353	< 0.001
Trustworthiness	5.73	0.72	2.55	0.71	289.655	< 0.001
Purchase intention	5.21	0.63	2.12	0.65	403.877	< 0.001

### ERPs Results

**FIGURE 3:**
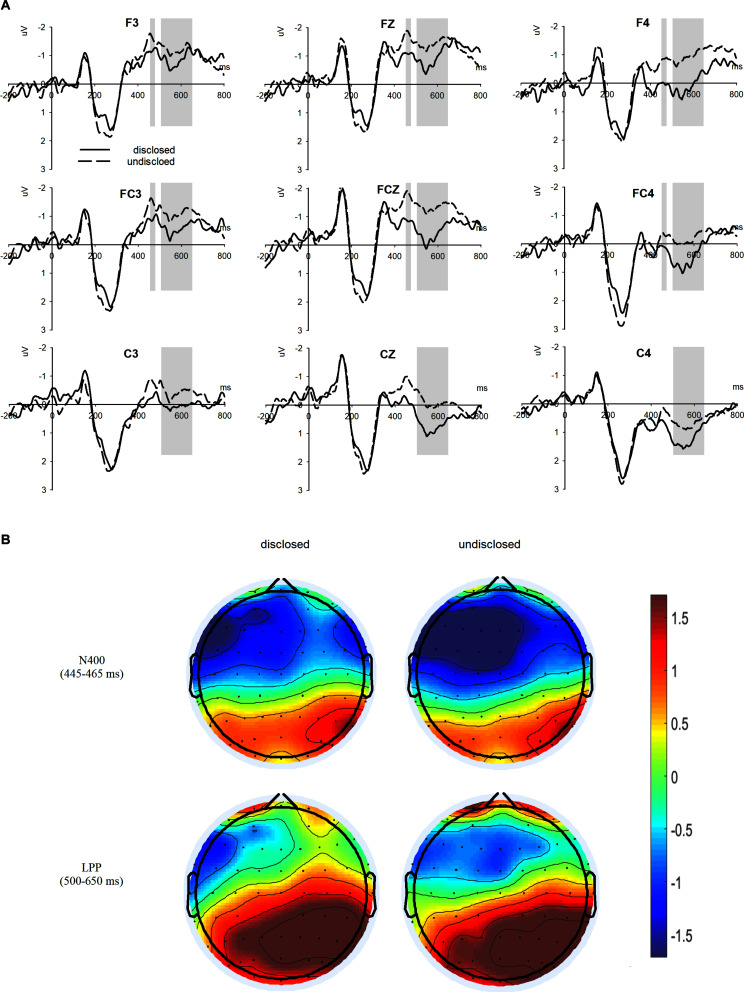
ERPs results. **(A)** the grand average waveforms at nine electrodes. **(B)** The topographic distributions of the waves of N400 and LPP.

As shown in Figure 3, the ANOVA on N400 amplitude showed that the main effect of disclosure was marginally significant [*F*(1, 32) = 3.515, *p* = 0.070, $\eta_{p}^{2}$ = 0.099]. A more negative N400 amplitude was elicited by the undisclosed condition (*M* = −1.504 μV, *SE* = 0.341) than by the disclosed condition (*M* = −0.866 μV, *SE* = 0.312). The main effect of electrodes was significant [*F*(1, 32) = 9.875, *p* < 0.001, $\eta_{p}^{2}$ = 0.236]. The interaction effect between disclosure and electrodes was not significant.

The ANOVA on LPP amplitude revealed a significant main effect of disclosure [*F*(1, 32) = 5.765, *p* = 0.022, $\eta_{p}^{2}$ = 0.153]. The LPP amplitude in the disclosed condition (M = −0.198 μV, *SE* = 0.034) was larger than that in the undisclosed condition (*M* = −0.852 μV, *SE* = 0.321). The main effect of electrodes was also significant [*F*(1, 32) = 14.670, *p* < 0.001, $\eta_{p}^{2}$ = 0.314]. The interaction effect between disclosure and electrodes was not significant.

## Discussion

Given its crucial role in aiding purchase decision-making, OCRs have become very popular among online consumers. Though a lot of effort has been devoted to investigating the impact of source credibility of OCRs on consumer decisions, few studies have explored the impact of reviewer’s disclosure of personal review record in this scenario. The present study is intended to investigate this issue. By engaging participants in virtual shopping tasks via the EEG method, the results may provide a nuanced understanding of consumers’ online decision processes.

Behaviorally, a higher purchase rate was observed in the disclosed condition than the undisclosed condition. Since the same product ratings were assigned to different experimental conditions, any differences in consumer decisions could not be attributed to product ratings. The finding that participants were more likely to buy a T-shirt when they noticed that the personal review record was disclosed was consistent with previous studies on reviewer identity disclosure and purchase decisions (Cox et al., 2009; Xie et al., 2011). This choice phenomenon could be explained from the perspective of signaling theory (Akerlof, 1970; Kirmani and Rao, 2000). Different parties in a market interaction often have different amount of information. More specifically, though reviewers know the credibility of their reviews, consumers are not fully informed of the credibility of the reviews. Therefore, quality signaling is used to communicate hidden or limited quality information to potential consumers to overcome information asymmetry (Wells et al., 2011). By disclosing the personal review record in the online environment, the reviewer sent a signal to the potential consumers that his reviews were open to social scrutiny and that his personal identity was identifiable. Consequently, participants inferred a higher level of perceived trustworthiness (a sub-dimension of credibility) when the personal review record was disclosed than when it was not (Chesney and Su, 2010), as was indicated by the rating results collected after the EEG experiment. A higher level of perceived credibility further gave rise to a higher purchase likelihood in the disclosed (vs. undisclosed) condition, which coincided with prior research (Forman et al., 2008; Xie et al., 2011; Priester and Petty, 2016).

Moreover, less time was spent making decisions in the disclosed condition (vs. undisclosed condition). Previous studies have revealed that task completion times (i.e., RTs) are positively correlated with task difficulty and cognitive load, as the more difficult a task is, the more RT it takes to complete (Sweller, 1988; Wang et al., 2015). Consistent with previous studies, we found longer RTs in the undisclosed condition than the disclosed condition, which indicated that less cognitive effort was required for participants’ decision making in the disclosed condition (vs. the undisclosed condition). Compared to the undisclosed condition, the disclosed condition was more favorable to the participants, which promoted decision making.

In line with the behavioral pattern, an attenuated N400 component and an increased LPP component were observed in the disclosed condition (vs. the undisclosed condition). Dozens of studies have shown that N400 is related to semantic conflict (Steffensen et al., 2008; Kutas and Federmeier, 2011). However, recent research in neuromarketing has found that N400 and N400-like components might serve as indicators of cognitive and emotional conflict (Steffensen et al., 2008; Chen et al., 2010; Wang et al., 2015; Shan, 2016). For example, Chen et al. (2010) defined a conformity condition whereby a consumer buys a product when presented with consistently positive reviews and does not buy a product when presented with consistently negative reviews, and a counter-conformity condition whereby a consumer does not buy a product when presented with consistently positive reviews and buys a product when presented with consistently negative reviews. The authors found that the counter-conformity condition evoked a larger N400-like component than the conformity condition, suggesting that participants experienced stronger cognitive and emotional conflicts when making a counter-conformity purchase decision. The higher the level of conflict, the larger the N400 amplitude. Furthermore, Wang et al. (2015) noted that products presented under conflictive conditions (high rating and low sales, and low rating and high sales) evoked larger N400 amplitudes than those presented under consistent conditions (high rating and high sales, and low rating and low sales), which suggested that the conflictive conditions led to more cognitive and emotional conflict and required more cognitive control. In the current study, as evidenced by the trustworthiness ratings collected after the EEG experiment, the invisibility of personal review record induced a higher level of uncertainty about the reviewer’s identity and a lower level of credibility, which might arouse heightened conflict processing. As a result, the enlarged N400 component observed in the undisclosed condition (vs. the disclosed condition) suggests that the undisclosed condition leads to more cognitive and emotional conflict and requires more cognitive control.

LPP is a well-established ERPs component that is indicative of evaluation and categorization and sensitive to both explicit and implicit categorization (Ito and Cacioppo, 2000; Wang et al., 2015). Importantly, recent research on neuromarketing has uncovered a close association between LPP component and evaluative categorization at the late cognitive processing stage (Wang et al., 2015; Jin et al., 2017). In the current study, the discrepancy in LPP amplitudes suggests that the disclosed condition is classified as being more favorable to the participants. Category similarity has been found to be crucial during evaluative categorization. More attentional resources will be allocated and a larger LPP component will be elicited when the presented category is close to the favorable target category (Fu et al., 2019). In the current study, participants formed expectations about the favorable characteristics of a reviewer and adopted the disclosed condition as a criterion category because it signaled high source credibility. During the task, the presented personal review record cue was automatically compared to the criterion. Hence, amplitudes of LPP were found to be larger in the disclosed condition than in the undisclosed condition. The behavioral data also support this interpretation. On the one hand, participants showed a higher purchase rate and faster reaction time in the disclosed condition. On the other hand, participants rated higher perceived trustworthiness in the closed condition. Taken together, the LPP result might imply the consumers’ evaluative categorization process at the late cognitive processing stage.

### Theoretical and Practical Implications

Theoretically, this research represents one of the first studies to contribute insight into the role of personal review record in consumer behavior. Though prior research has devoted a lot of attention to reviewer’s self-disclosure, the disclosure of personal review record has remained underexplored. This research bridges the gap in the literature. Moreover, this research complements extant literature on signaling theory. To the best of our knowledge, this is the first study to conceptualize the disclosure of personal review record as a signal that could be used to assure the consumers of the reviewer’s credibility. Specifically, compared with a reviewer who does not disclose his (or her) personal review record, a reviewer who does leads to a higher level of perceived trustworthiness and greater willingness to purchase the reviewed product. Finally, our findings also extend extant research on neuromarketing. By combining behavioral and ERPs approaches, this study provides a nuanced understanding of how self-disclosure of personal review record influences consumer information processing and decision-making.

Our study also has practical implications for online shopping platform operators, marketers, and consumers. The findings of the present study suggest that the disclosure of reviewers’ personal review record could enhance the perceived credibility of product reviews, which will ultimately influence the persuasiveness of reviews and lead to an increase in purchase rate. From this point of view, this study may serve as a reference for online information presentation. An absence of personal review record on e-commerce platforms (e.g., Taobao.com) might significantly reduce the perceived credibility of reviews, which might in turn lower consumers’ purchase intentions. Alternatively, reviewers may disclose identity information to enhance others’ perceived credibility of their reviews. Hence, it’s highly recommended that e-commerce platforms establish mechanisms that encourage reviewers to disclose their identity information to potential consumers who turn to reviews for shopping guidance, which will finally promote the development of electronic commerce.

### Limitations and Future Research

Although this study offers some interesting findings, there are some limitations worth highlighting for future research. First, this study is focused on only one type of product (T-shirts). We chose T-shirts as the products because most people are familiar with T-shirts and they have been frequently used in prior neuromarketing research (Yokoyama et al., 2014; Shang et al., 2017). T-shirts are relatively inexpensive and belong to utilitarian products. A replication of the study based on a wider range of products (e.g., expensive or hedonic products) could be conducted to generalize the findings of the present study. Second, the results only showed a marginally significant effect of disclosure on N400 when we selected six electrodes (F3, FZ, F4, FC3, FCZ, and FC4) for N400 analysis. We surmise that it might not be due to the sample size because the power analysis suggested that the sample size of the current study met the requirement. In fact, though we didn’t find an interaction between disclosure and electrodes on N400, the effect of disclosure on N400 turned out to be statistically significant (*p* = 0.043) when we selected four electrodes in the middle and right scalp regions (FZ, F4, FCZ, and FC4) for N400 analysis. Consequently, we speculate that the conflict information may be processed mainly in the middle and right scalp regions in the present study. Further studies are required to confirm this point. Third, as an exploratory study, only information cues were used to signal if the personal review record was disclosed in order to eliminate possible confounding factors. Future research may adopt more vivid presentation of personal review record.

## Conclusion

In summary, through this study we investigated the effect of reviewers’ self-disclosure of personal review record on consumers’ purchase decision making and the underlying neural substrates. Behaviorally, the disclosed condition led to higher purchase rates and shorter reaction times. The electrophysiological results showed an attenuated N400 and an enlarged LPP for the disclosed condition (vs. the undisclosed condition), indicating that the disclosed condition resulted in less conflict and more positive evaluations. In line with source credibility theory and signaling theory, the results suggest that the disclosure of personal review record could enhance the perceived trustworthiness of reviews and help consumers make purchase decisions. The findings of the current research contribute to the self-disclosure literature by uncovering the effect of personal review record.

## Data Availability Statement

The raw data supporting the conclusions of this article will be made available by the authors, without undue reservation.

## Ethics Statement

The studies involving human participants were reviewed and approved by the Internal Review Board of the Laboratory of Neuromanagement and Decision Neuroscience of Guangdong University of Technology. The patients/participants provided their written informed consent to participate in this study.

## Author Contributions

JL and HF conceived and designed the study, interpreted the data, and drafted the manuscript. JL collected and analyzed the data. JL, ZM, HF, WW, LS, and KL reviewed and edited the manuscript. HF administered the project. All authors contributed to the article and approved the submitted version.

## Conflict of Interest

The authors declare that the research was conducted in the absence of any commercial or financial relationships that could be construed as a potential conflict of interest.
